# Numerical Investigation of Short-Channel Effects and RF Performance in Top-Gate In_2_O_3_ Thin-Film Transistors

**DOI:** 10.3390/mi17050567

**Published:** 2026-05-02

**Authors:** Hanbo Xu, Mingyang Zhu, Zeen Fang, Lei Zhang

**Affiliations:** College of Mechanical and Electrical Engineering, Central South University, Changsha 410083, China; 8203231004@csu.edu.cn (H.X.); 8203230513@csu.edu.cn (M.Z.); 8203231006@csu.edu.cn (Z.F.)

**Keywords:** indium oxide (In_2_O_3_), top-gate, down-scaling, short-channel effect, MOSFET

## Abstract

Indium oxide (In_2_O_3_) has recently emerged as a promising semiconductor for advanced electronics due to its high electron mobility and wide bandgap. In this article, the lateral scaling characteristics of top-gate In_2_O_3_ thin-film transistors (TFTs) featuring a 1.5 nm thick channel and a 7 nm thick HfO_2_ gate dielectric are investigated by two-dimensional device simulation. The analysis covers short-channel effects, DC characteristics, transconductance behavior, and small-signal radio frequency (RF) metrics across a gate-length (*L_G_*) range of 20 nm to 700 nm. Simulation results identify a critical gate length near 100 nm for the transition from long-channel to short-channel behavior. For *L_G_* ≤ 100 nm, pronounced short-channel effects emerge, featuring a significant negative *V_TH_* shift and a drain-induced barrier lowering (DIBL) coefficient up to ~130 mV/V. A non-classical gm scaling behavior is observed, where *g_m___max_* initially increases with *L_G_*, then remains within a narrow range and eventually evolves toward the conventional long-channel trend. Further analysis of the lateral electric field distribution, field-dependent mobility, and transconductance efficiency indicates that this behavior originates from a crossover between short-channel field-assisted transport and gate-controlled channel modulation. The devices show strong RF potential, with *f_T_* and *f_max_* reaching 124.32 GHz and 157.64 GHz, respectively, at *L_G_* = 20 nm. The high-mobility In_2_O_3_ channel leads to a less distinct *f_T_* scaling transition from the classical 1/*L*^2^*_G_* dependence to the short-channel 1/*L_G_* dependence, while *f_max_* scaling evolves through different regimes governed by capacitance-related limitations, intrinsic transport enhancement, and short-channel non-idealities. This work provides physical insight into the lateral scaling behavior of ultrathin top-gate In_2_O_3_ TFTs and highlights their potential for high-frequency and power-dense applications.

## 1. Introduction

Metal oxide semiconductors have attracted extensive interest for next-generation electronics because they combine several attractive features, including wide bandgap, process compatibility with large-area and low-temperature fabrication, and the potential for high electron transport in ultrathin channels [1,2]. Compared with conventional semiconductor platforms, oxide semiconductors are also well-suited for transparent, flexible, and back-end-of-line-compatible device applications. Within this material family, In_2_O_3_ is particularly attractive because it can provide relatively high electron mobility and high current-driving capability even in atomically thin channels, making it a strong candidate for aggressively scaled thin-film transistors and high-frequency devices [3,4]. With the rapid development of atomic layer deposition (ALD), atomically thin In_2_O_3_ channels have become increasingly feasible, which has stimulated growing interest in ultrathin In_2_O_3_ FETs for both logic-oriented and radio frequency (RF)-oriented applications [5,6].

Currently, ALD In_2_O_3_ back-gate FETs stand out due to their excellent conformality and uniformity on three-dimensional structures enabled by the ALD process, as well as high electron mobility, high on-current, and remarkable reliability [7]. Back-gate-structured In_2_O_3_ field-effect transistors have achieved significant progress. Notably, back-gate In_2_O_3_ FETs have achieved record-high drain currents exceeding 2000 mA/mm with aggressive channel length scaling down to 40 nm [8] and have demonstrated high-performance operation even with ultrathin channel thicknesses below 1 nm [9]. However, from the perspective of practical device integration, top-gate In_2_O_3_ FETs have attracted increasing attention, although early top-gate devices suffered from high off-state current, negative threshold voltage, and difficulty in achieving complete turn-off. Nevertheless, recent studies have demonstrated that the inherent defects in top-gate In_2_O_3_ FETs can be significantly mitigated through appropriate interface engineering. For instance, a recent work successfully developed high-reliability In_2_O_3_ top-gate thin-film transistors via in situ surface energy engineering in the ALD process, revealing that the performance limitations previously associated with the top-gate structure can be markedly improved [10]. Significantly, In_2_O_3_ TFTs subjected to an optimized oxygen plasma treatment duration of 3 s exhibited significant improvements in electrical characteristics, including a high-field-effect mobility of 84.3 cm^2^/V·s, a steep subthreshold swing of 76.8 mV/dec, etc. [10].

From these perspectives, top-gate In_2_O_3_ devices are of particular interest because they combine the intrinsic transport advantages of ultrathin In_2_O_3_ with a gate-stack configuration more relevant to practical device integration. Owing to its high output current density, the top-gate In_2_O_3_ transistor is highly suitable for radio frequency (RF) power devices. Recent demonstrations of top T-gate BEOL-compatible In_2_O_3_ FETs have achieved a record *fₘₐₓ* of 70 GHz, along with high current density and transconductance, showcasing their potential for future RF and millimeter-wave applications [11]. Furthermore, top-gate In_2_O_3_ transistors show excellent scalability and high current drive, highlighting their potential for high-speed applications [12]. However, in the study of RF power transistors, as device dimensions scale down, short-channel effects become more pronounced. Therefore, understanding short-channel behavior in top-gate In_2_O_3_ transistors has become increasingly important.

Herein, motivated by recent advances in In_2_O_3_ top-gate TFTs, a systematic numerical study on the short-channel effects in these devices is presented to demonstrate their potential for high-frequency operation. This study is conducted using a two-dimensional device simulation tool (Silvaco Atlas 2020), and the short-channel effects as well as the relationship between the *f_T_* and the *L_G_* are analyzed.

## 2. Simulation

### 2.1. Calibration

To establish experimentally grounded material and transport parameters for the TCAD simulation, the In_2_O_3_-related parameter set was first calibrated using a previously reported back-gate In_2_O_3_ FET [13]. This calibration step was not intended to equate the back-gate architecture with the target top-gate device studied here. Instead, it served as an experimentally accessible starting point for determining the effective In_2_O_3_ transport and interface-related parameters used in the subsequent top-gate simulation.

In the present work, the term “calibration” refers to the extraction of effective material, transport, and interface-related parameters for the TCAD framework by matching the simulated output and transfer characteristics to those of a reported experimental In_2_O_3_ device. The device geometry and stack information available from the reference were fixed first, while a limited set of parameters, including the low-field mobility, effective channel doping level, interface-trap-related quantities, fixed interface charge, and saturation velocity, were adjusted within physically reasonable ranges. The fitting targets were the drain current magnitude, threshold voltage trend, and overall shapes of the output and transfer characteristics. The calibrated parameter set was then transferred to the top-gate device platform for the scaling analysis in this work. The top-gate device investigated in this work was then constructed independently using the gate-stack geometry, interface conditions, and contact configuration defined in this study. The reported back-gate device was selected as the primary calibration anchor because its short-channel dimensions are more relevant to the present scaling-oriented analysis than the currently available long-channel top-gate experimental reports.

Although recent top-gate In_2_O_3_ TFT studies provide valuable experimental guidance, the available top-gate dataset used in Ref. [10] corresponds to a much longer channel length (*L_G_* = 20 μm) and was therefore not adopted as the primary calibration target for the present short-channel scaling study.

**Figure 1:**
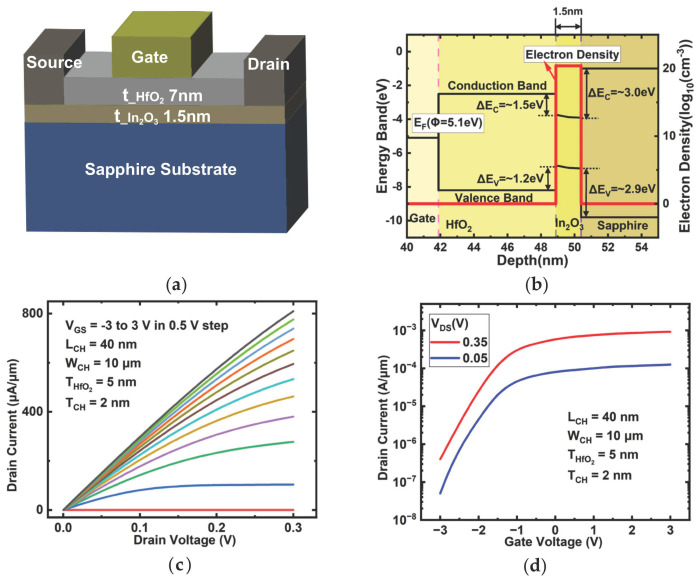
(**a**) Schematic device structure of the simulated top-gate In_2_O_3_ FET. (**b**) Energy band diagram and electron density profile with *V_G_* = 0 V. (**c**,**d**) Calibration of the simulated output and transfer characteristics of a back-gate In_2_O_3_ FET with Ref. [13]. In (**c**), different colored curves represent V_GS_ values from −3 V to 3 V with a step of 0.5 V, increasing from bottom to top. (fitting units are kept consistent with the reference).

The parameters listed in Table 1 were adjusted to obtain the calibration results shown in Figure 1c,d. Compared with the calibration plots in Ref. [13], although an exact point-by-point match is not expected in a device-level TCAD framework, the calibrated model reproduces the main current level, threshold trend, and overall output and transfer characteristics of the reference device. The extracted parameter set (Table 1) was subsequently applied to the top-gate device platform used in this work for scaling analysis.

### 2.2. Device Structure

The three-dimensional structure of the simulated device is depicted in Figure 1a. For the target top-gate device platform studied in this work, a sapphire substrate was selected as an electrically insulating supporting substrate. This choice was made to reduce substrate-induced parasitic coupling and to provide a stable platform for analyzing the intrinsic influence of gate-length scaling on short-channel electrostatics and RF metrics [14,15].

The purpose of the present study is not to reproduce a specific previously reported substrate stack one-to-one [10,13], but to investigate the scaling behavior of a clearly defined top-gate In_2_O_3_ device platform. For comparison, an additional substrate sensitivity result is provided in the Supplementary Materials [10,13].

Owing to its multiple advantages as a gate dielectric for In_2_O_3_ FETs, including a high dielectric constant (high-κ) for enhanced electrostatic control and a reported low interface trap density in HfO_2_/In_2_O_3_ gate stacks [16,17], a 7 nm HfO_2_ layer was employed as the gate oxide. The gate, source, and drain electrodes were formed of nickel, with a work function of 5.1 eV. The source and drain were defined as ohmic contacts.

**Figure 2:**
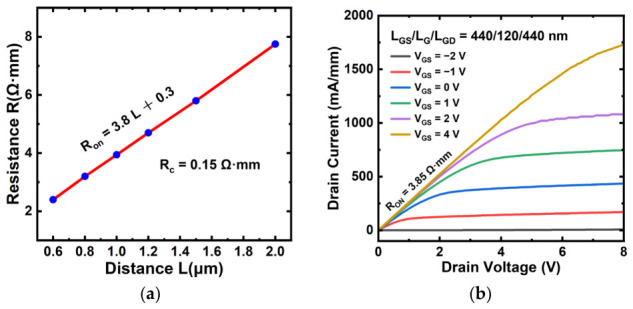
(**a**) Variation in resistance with electrode distance L in TLM model to extract contact resistance R_c_. (**b**) Output characteristic of simulated In_2_O_3_ FET with L_GS_:L_G_:L_GD_ = 440:120:440 nm.

Figure 2a presents resistance as a function of the electrode spacing deduced from a transmission line model (TLM) test structure simulated by our top-gate In_2_O_3_ FET with the gate removed. As depicted in Figure 2a, a low contact resistance (*R_c_*) of 0.15 Ω·mm was derived by TLM. The ultra-low contact resistance of 0.15 Ω·mm indicates well-configured ohmic contact parameters. For devices of all gate lengths in this work, the source-to-gate and drain-to-gate edge distances (i.e., the gate–source spacing *L_GS_* and gate–drain spacing *L_GD_*) vary with gate length. This is because the source-to-drain distance *L_SD_* is fixed at 1 μm for all devices, so varying the gate length inevitably alters *L_GS_* and *L_GD_*, representing a non-standard scaling approach. Since the gate is centered, the relationship can be derived as *L_GS_* = *L_GD_* = (*L_SD_* − *L_G_*)/2.

### 2.3. Physical Model

For the top-gate In_2_O_3_ FET, the non-ideal HfO_2_/In_2_O_3_ gate interface was modeled by introducing acceptor-like interface traps and fixed positive charges. The trap level was set at approximately 0.5 eV below the conduction band minimum, with a trap density of 3 × 10^11^ cm^−2^, electron and hole capture cross-sections of 1 × 10^−14^ cm^2^, and a degeneracy factor of 2. In addition, a fixed positive interfacial charge density of 1 × 10^10^ cm^−2^ was included. These quantities were used as calibration-guided effective interface parameters based on literature-reported experimental characteristics [18].

By contrast, the In_2_O_3_/sapphire bottom interface was treated as electrically passive in the present top-gate framework, because the gate electrostatics are primarily governed by the HfO_2_/In_2_O_3_ top interface.

Owing to the ultra-high channel doping concentration (2.5 × 10^20^ cm^−3^) and the ultrathin 1.5 nm In_2_O_3_ body, the channel operates in a highly degenerate carrier concentration regime. Therefore, Fermi–Dirac carrier statistics were enabled in the Atlas simulation.

The parallel field transport was described using the Caughey–Thomas field-dependent mobility (FLDMOB) formulation, because it provides an effective and physically interpretable representation of mobility degradation and carrier-velocity saturation under the strong lateral electric fields relevant to the present scaling study. In the FLDMOB model, expressed in Formula (1), *μ_n_*(*E*) is the field-dependent electron mobility, the carrier drift velocity is defined as *v_n_* = *μ_n_*(*E*)∙*E*, and *E* is the parallel electric field:

(1) $$\mu_{n}(E)=\frac{\mu_{0}\cdot v_{sat}}{v_{sat}+\mu_{0}\cdot \left|E\right|}$$

here, the adopted low-field mobility (*μ*_0_ = 120 cm^2^/V·s) and electron saturation velocity (*v_sat_* = 1 × 10^7^ cm/s) were selected within experimentally reported ranges for In_2_O_3_-based devices [19] and were further refined during the calibration procedure. In this sense, the FLDMOB-based transport description is used here as a transport representation for trend analysis rather than as a complete non-equilibrium transport formalism.

**Figure 3:**
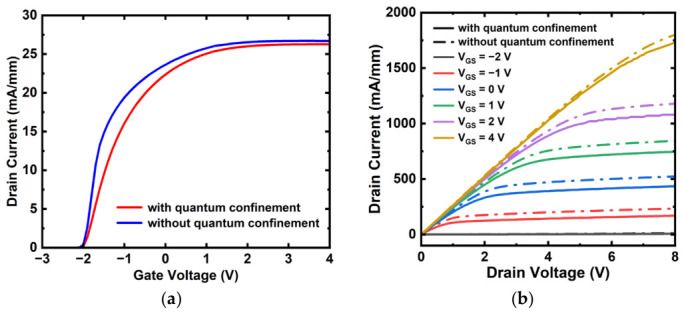
(**a**) Transfer and (**b**) output characteristics of the top-gate In_2_O_3_ TFT in this work with and without quantum confinement at *L_G_* = 120 nm.

Considering that the 1.5 nm thick In_2_O_3_ channel is already atomically thin, quantum confinement is expected to affect the electrostatic and carrier-distribution behavior. Therefore, a density-gradient-based quantum correction was included in the TCAD framework as an approximate quantum confinement treatment on top of the field-dependent mobility model. Figure 3a,b demonstrate the transfer and output characteristics of the top-gate In_2_O_3_ TFT in this work with and without quantum correction at *L_G_* = 120 nm. It can be observed that when the quantum confinement effect is included, the drain current decreases in both the linear and saturation regions, leading to an overall downward shift of the curves. In addition, the slope in the linear region is reduced. These changes are mainly attributed to the fact that in nanoscale devices, the quantum confinement effect shifts the peak of the carrier distribution away from the interface and causes an effective band broadening, thereby modifying the electrical behavior of the device.

The present transport simulation remains based on a drift-diffusion-type framework with field-dependent mobility and quantum correction. Although quasi-ballistic effects may become relevant in atomically thin In_2_O_3_ films under aggressive scaling, they were not explicitly included in the present TCAD framework and are therefore regarded as a limitation of this work.

In addition, impact ionization was included through the Selberherr model to avoid neglecting high-field carrier-generation effects under large *V_DS_* and short-channel conditions. Its influence is considered as a supporting high-field correction in this work rather than as an independent focus of this study.

The SRH carrier lifetime was set to 10 ns as a calibration-guided simulation parameter used to represent defect-assisted recombination within the present TCAD framework. The selected lifetime mainly serves as an effective recombination parameter within the calibrated model rather than a directly measured material lifetime [20]. The transport analysis in this work focuses on electron-dominated conduction in n-type In_2_O_3_ TFTs and is based on a drift-diffusion-type formulation. Direct source-to-drain tunneling, gate leakage, hydrodynamic transport, and self-heating effects were not explicitly included. These effects are expected to become more relevant under more aggressive scaling or higher-field operating conditions and are therefore identified as limitations of the current framework and important topics for future work.

After all physical models have been applied, the device band diagram is exported from the software. The extracted corresponding energy band diagram and the electron density profile under the gate are presented in Figure 1b and are consistent with the In_2_O_3_/HfO_2_ interface band characteristics experimentally characterized by Tung-Yuan Yu et al. [21]. Specifically, the output characteristics of the simulated device with *L_GS_*:*L_G_*:*L_GD_* = 440:120:440 (units in nm) are presented in Figure 2b, which are in good agreement with the typical output characteristics of a conventional MOS-FET.

## 3. Simulation Results and Discussion

### 3.1. Direct Current Characteristics

**Figure 4:**
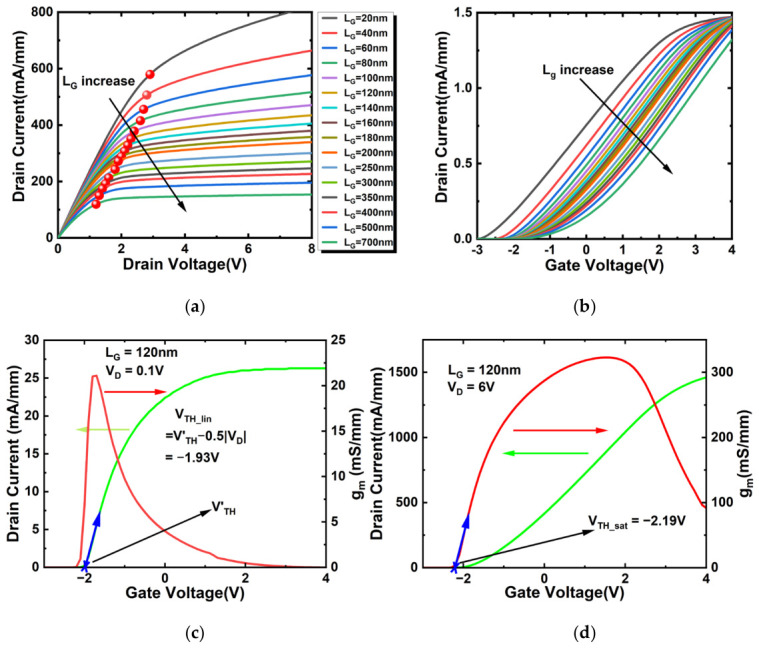
(**a**) Output characteristics of the top-gate In_2_O_3_ FETs with different gate lengths (*L_G_*); (**b**) transfer characteristics of the top-gate In_2_O_3_ FETs at different gate lengths, and transfer characteristics and transconductance of In_2_O_3_ FET with *L_G_* = 120 nm with drain voltage biased at (**c**) 0.1 V and (**d**) 6 V.

Figure 4a presents the output characteristics at different gate lengths (*V*_GS_ = 0 V) and Figure 4b shows the transfer characteristics in the saturation region (*V*_DS_ = 6 V). As device dimensions scale down, the effective channel length decreases, leading to an increase in output current that no longer exhibits saturation. As the gate length (*L*_G_) decreases, the on-resistance (*R*_on_) is reduced due to the lateral scaling of the device. Under a constant gate bias, the electric field strength in the short channel becomes higher compared to that in devices with longer L_G_. This enhanced electric field accelerates the drift velocity of electrons in the channel, consequently leading to an increase in the saturation drain current (*I*_Dsat_). This finding is consistent with previous numerical studies on H-terminated diamond field-effect transistors (H-diamond FETs) reported in [20]. It is noteworthy, however, that the R_on_ of the top-gate In_2_O_3_ FET is evidently lower than that of the H-terminated diamond FET (H-diamond FET). This is attributed to the relatively high electron mobility (~120–140 cm^2^/(V·s)) in ultrathin indium oxide, which is significantly higher than the hole mobility in H-diamond.

The high electron mobility renders the intrinsic saturation drain voltage (*V_Dsat_*) of the top-gate In_2_O_3_ FET responsive to the increase in lateral electric field upon device scaling. As observed in Figure 4a, the position of the knee voltage varies with gate length and does not align vertically. This misalignment becomes particularly pronounced when entering the short-channel regime (*L_G_* < 100 nm), where a further reduction in gate length leads to significant shifts in the knee voltage. Compared with the intrinsic *V_Dsat_* of 3–4 V for devices with *L_G_* > 100 nm, that of short-channel devices even exceeds 6 V. The saturation voltage of short-channel devices warrants reexamination, as the continual increase in drain current with *V_d_* rising from 4 to 6 V constitutes a manifest indication of short-channel effects. On the other hand, the total contact resistance, comprising the source resistance (*R_S_*) and drain resistance (*R_D_*), is approximately 0.30 Ω·mm. This value is significantly lower than that reported in Ref. [20], and thus the contact resistance does not substantially degrade the output current.

For short-channel top-gate In_2_O_3_ FETs, while a relatively large intrinsic *V_Dsat_* may pose challenges, it can also be indicative of certain favorable device characteristics under specific conditions [22]. The high electron density of In_2_O_3_ is an advantage, facilitating high transconductance (*g_m_*) and cutoff frequency (*f_T_*) [23], yet it is accompanied by a relatively low breakdown field strength for the device [24]. The simulation predicts an extremely high intrinsic *V_Dsat_*; however, in practical applications, self-heating and breakdown limitations must be considered, which will be the focus of future work.

The top-gate In_2_O_3_ FET with a gate length of 120 nm was biased in the on-state. Figure 4c shows the linear region (*V_DS_* = 0.1 V) transfer characteristics along with the extracted *g_m_* curve. From this, the linear threshold voltage (*V_TH_lin_*) is extracted based on the formula *V_TH_lin_* = *V_TH_* − 0.5*|V_DS_|*, where *V_TH_* is determined as the gate-voltage intercept of the tangent line drawn at the point of maximum slope on the transfer curve. Figure 4d displays the saturation region (*V_DS_* = 6 V) transfer characteristics and the extracted *g_m_* curve, from which the saturation threshold voltage (*V_TH_sat_*) is determined. Here, *V_TH_sat_* is defined as the gate-voltage intercept of the tangent line drawn at the point of maximum slope on the *g_m_* curve.

**Figure 5:**
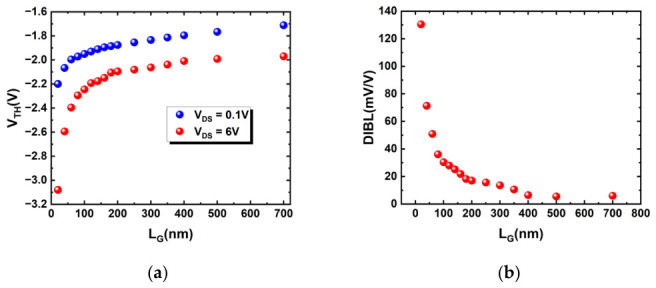
(**a**) *V_TH_* variation with gate length with *V_DS_* = 0.1 V and 6 V; (**b**) DIBL versus *L_G_* of the devices, extracted at constant *I_D_* = 0.1 mA/mm with *|ΔV_DS_|* = 5.9 V.

As shown in Figure 5a, with a gate oxide thickness of 7 nm and gate length greater than 150 nm, the threshold voltage in the linear region remains nearly stable, ranging from −1.9 V to −1.8 V, while in the saturation region, it stabilizes between approximately −2.1 V and −2.0 V. A clear trend observed in Figure 5a is that progressively decreasing *L_G_* below 150 nm results in a correspondingly larger shift in *V_TH_*. Notably, when the gate length is scaled down below 100 nm, the absolute shift in threshold voltage (|*ΔV_TH_*|) for both the linear and saturation regions exhibits a near-linear dependence on the inverse gate length (1/*L_G_*). This dependence is consistently more pronounced in the saturation region than in the linear region.

Figure 5b elucidates several potential mechanisms underlying this behavior. Drain-induced barrier lowering (DIBL) refers to the phenomenon in short-channel devices where a high *V_DS_* effectively lowers the potential barrier between the source and the channel, enabling the device to turn on at a lower gate voltage. Quantitatively, it is defined as the ratio of the threshold voltage shift to the drain voltage difference (DIBL = |*ΔV_TH_|*/|*ΔV_DS_*|). In long-channel devices, the channel potential is governed exclusively by the gate electrode through the vertical electric field. As the channel length shortens, the lateral electric field generated by the high drain voltage significantly affects the barrier height at the source end [25]. Therefore, for gate lengths greater than 150 nm, the channel is sufficiently long such that the influence of the drain voltage is negligible. Consequently, the *V_TH_* is dominated by and remains stable with respect to the gate voltage. In the saturation region (*V_DS_* = 6 V), the high drain voltage establishes a strong electric field at the drain terminal. The field lines extend toward the source, effectively lowering the gate voltage required for turn-on. Compared to the linear region (*V_DS_* = 0.1 V), this mechanism manifests as a negative shift in the *V_TH_*. In shorter channels, the influence of the drain on the source becomes stronger. Consequently, a more negative gate voltage is required to compensate for this drain-induced assistance to achieve device turn-off, leading to an increased magnitude of the negative *V_TH_* shift. From another perspective, the drain voltage in the linear region is very low (*V_DS_* = 0.1 V), resulting in a weak lateral electric field. In contrast, the high drain voltage in the saturation region (*V_DS_* = 6 V) generates a much stronger lateral field, whose effect on lowering the source-end potential barrier is substantially greater than that in the linear region. When the channel becomes extremely short (*L_G_* < 100 nm), the influence of the drain becomes predominant. Its dependence on the gate length converges to a stable, linear trend, indicating that the device has entered a pronounced short-channel regime where the *V_TH_* is strongly modulated by the drain voltage. As observed in Figure 5b, when the gate length is scaled down below 100 nm, the DIBL effect becomes significant, with its coefficient increasing from approximately 6 mV/V in the long-channel regime to about 130 mV/V.

**Figure 6:**
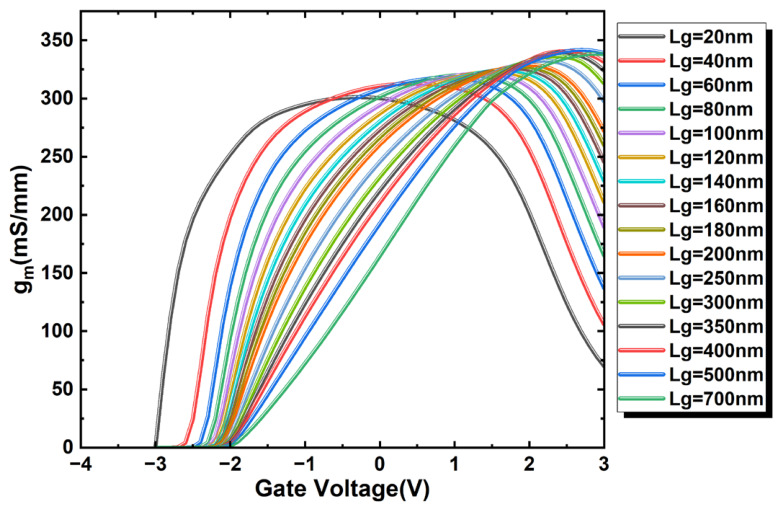
Dependence of *g_m_* on gate voltage for devices with different gate lengths in the saturation region (*V_DS_* = 6 V).

Figure 6 presents the variation in saturation region *g_m_* with *V_GS_* for a fixed source-to-drain spacing (*L_SD_*). Following the maximum slope method for *V_TH_sat_* extraction described previously, it is observed that the absolute value of the saturation threshold voltage (|*V_TH_sat_*|) decreases progressively with increasing *L_G_*. Consequently, a less negative gate voltage is required to turn on the device. This is because a longer gate length increases the gate area, thereby enhancing the control of the channel potential by the vertical electric field. Furthermore, as analyzed above, the reduction in |*V_TH_sat_*| is directly linked to the significant attenuation of the DIBL effect.

Although the simulated devices exhibit strong current-driving capability, the transconductance does not increase indefinitely with current density. This is because *g_m_* is determined not only by the drain-current level but also by how efficiently the gate voltage modulates the channel charge and carrier velocity. In the present top-gate In_2_O_3_ TFTs, the *g_m_* behavior is jointly influenced by the electrostatic coupling of the 7 nm HfO_2_ gate stack, the highly doped ultrathin channel, short-channel drain field effects, and velocity-saturation-related transport. As a result, a high current density does not automatically translate into a proportionally high *g_m_*. This indicates that the relatively moderate *g_m_* is mainly limited by the efficiency of gate-controlled charge modulation and high-field-transport degradation, rather than by the available carrier density alone.

From a device design perspective, several routes can be expected to further improve *g_m_*: reducing the effective oxide thickness to strengthen gate control and increase channel-charge modulation efficiency; improving the HfO_2_/In_2_O_3_ interface quality and reducing interface traps to enhance gate coupling and suppress parasitic degradation of transconductance; and lowering source/drain-access resistance while optimizing contact/gate alignment to further improve the effective *g_m_* observed in RF operation. Hence, the present results suggest that *g_m_* in top-gate In_2_O_3_ TFTs remains closely tied to electrostatic design and interface/contact engineering, rather than being determined by carrier density alone.

**Figure 7:**
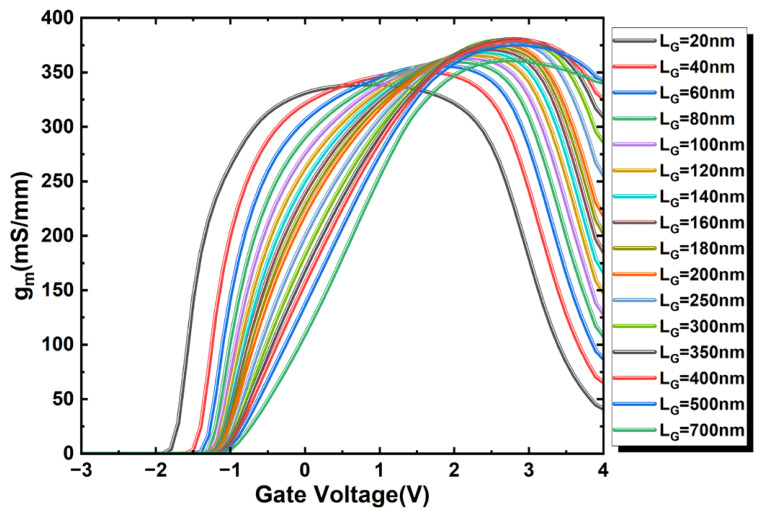
*g_m_* versus *V_GS_* curves for top-gate In_2_O_3_ TFTs with various gate lengths after reducing the HfO_2_ thickness from 7 nm to 4 nm.

To evaluate the possible *g_m_* improvement through enhanced gate coupling, the 4 nm HfO_2_ case was selected as a representative thinner-dielectric condition. As discussed previously, Figure 7 presents the *g_m_*–*V_GS_* characteristics of top-gate In_2_O_3_ TFTs with different gate lengths after the HfO_2_ thickness is reduced from 7 nm to 4 nm. Compared with the 7 nm HfO_2_ case (Figure 6), the transconductance of the short-gate-length devices increases from approximately 340 mS/mm to 375 mS/mm. This indicates that adopting a thinner gate dielectric can enhance gate control capability and thereby increase *g_m_*. However, further reduction of the gate dielectric thickness may also increase gate leakage and electric field stress, so the improvement in gm should be balanced against reliability considerations.

**Figure 8:**
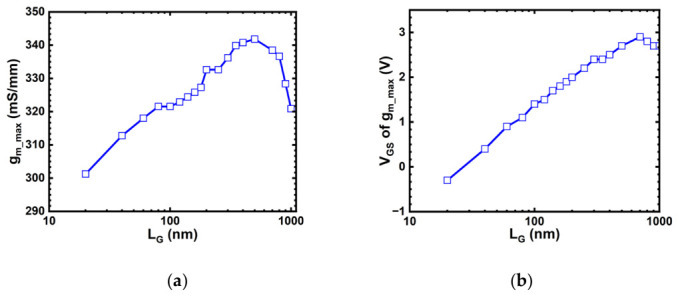
(**a**) Maximum *g_m_* and (**b**) its *V_GS_* position versus *L_G_*.

Concurrently, it is noteworthy that the trend presented in Figure 8a, which is derived from the maximum transconductance values of Figure 6, differs from that of conventional silicon-based MOSFETs:

(2) $$g_{m}\propto (\mu_{0}C_{G})(V_{GS}-V_{TH})/L_{G}$$

According to the well-established long-channel relationship, the maximum transconductance (*g_m_max_*) is generally expected to decrease with increasing *L_G_* [26]. However, the trend extracted from Figure 8a for the present top-gate In_2_O_3_ FETs is clearly non-classical: *g_m_max_* first increases with *L_G_*, then remains within a relatively narrow range of about 320–340 mS/mm over an intermediate gate-length range, and only at larger *L_G_* does it begin to recover the conventional decreasing tendency. This behavior suggests that the *g_m_max_* scaling in the ultrathin In_2_O_3_ channel is better interpreted as a crossover between different electrostatic and transport regimes rather than by the classical 1/*L_G_* scaling law alone.

In the very-short-channel regime, the channel behavior is strongly affected by drain-field-assisted transport and short-channel electrostatics, which weakens the direct applicability of the long-channel transconductance scaling picture. As *L_G_* increases from this regime, the gate gains stronger control over the channel potential and the channel-charge modulation becomes more effective, so *g_m_max_* can initially increase instead of decreasing. Over an intermediate *L_G_* range, the combined influence of strong gate coupling, high carrier concentration, and velocity-saturation-related transport causes *g_m_max_* to exhibit a plateau-like behavior rather than a pronounced geometrical scaling dependence. When *L_G_* becomes sufficiently large, the device gradually approaches a more classical drift-diffusion-dominated regime, and the expected reduction of *g_m_max_* with increasing gate length becomes more evident. Therefore, the non-monotonic/weakly saturated *g_m_max_* evolution observed in Figure 8a is more appropriately understood as a transport/electrostatic crossover specific to the highly doped ultrathin In_2_O_3_ channel rather than as a violation of MOSFET scaling physics.

**Figure 9:**
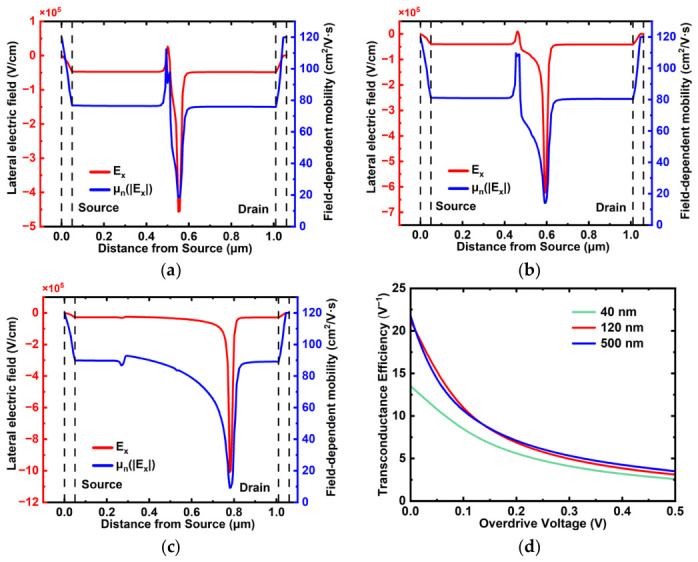
(**a**–**c**) Lateral electric field (*E_x_*) and field-dependent mobility (*μ_n_*(*|Ex|*)) calculated using the FLDMOB model (Formula (1)) as a function of distance from the source at V_GS_ = 2 V and V_DS_ = 6 V, extracted along a lateral cutline at the center of the In_2_O_3_ channel for different gate lengths: (**a**) *L_G_* = 40 nm, (**b**) 120 nm, and (**c**) 500 nm. (**d**) Transconductance efficiency versus overdrive voltage (*Vov* = *V_GS_* − V*_TH_*) for three gate lengths.

Figure 8b shows that the *V_GS_* position of *g_m_____max_* shifts progressively toward more positive values as *L_G_* increases. This trend is consistent with the evolution from stronger short-channel drain field assistance to stronger gate-controlled channel modulation. In shorter devices, the high drain field contributes more strongly to carrier injection and channel transport, so the condition for reaching peak transconductance can be achieved at a relatively less positive gate bias. As *L_G_* increases and the DIBL-related drain assistance is reduced, a larger gate overdrive is required to establish the charge modulation condition corresponding to maximum transconductance, leading to the observed positive shift of the *V_GS_* position of *g_m_____max_*. Taken together, Figure 8a,b indicate that channel length plays a key role in governing the transition from short-channel field-assisted transport behavior toward a more classical long-channel transconductance regime. This interpretation is further supported by the lateral electric field distribution, field-dependent mobility, and transconductance efficiency analysis presented in Figure 9.

To further clarify the non-classical evolution of *g_m_max_* with gate length, the lateral electric field distribution *E_x_*(*x*) and the corresponding field-dependent electron mobility *μ_n_*(*|Ex|*) were examined for representative devices with *L_G_* = 40, 120, and 500 nm under *V_GS_* = 2 V and *V_DS_* = 6 V, as shown in Figure 9a–c. In all cases, a pronounced high-field region is observed near the drain-side gate edge, accompanied by a clear local reduction in *μ_n_*, confirming that high-field transport and field-induced mobility degradation play an important role under saturation operation. The position of the mobility minimum follows the position of the maximum magnitude of the lateral electric field, indicating that the local carrier transport is strongly modulated by the lateral field distribution along the channel.

In addition, the transconductance efficiency *g_m_*/*I_D_* was evaluated as a function of overdrive voltage for different gate lengths, as shown in Figure 9d. The *g_m_*/*I_D_* curves decrease monotonically with increasing overdrive voltage for all devices, while the overall efficiency follows the trend of 500 nm > 120 nm > 40 nm. This indicates that longer-channel devices maintain more efficient gate control over channel charge, whereas the ultrashort-channel device suffers from stronger short-channel electrostatic degradation and drain field interference, which reduce the charge modulation efficiency. Therefore, the evolution of *g_m_max_* with *L_G_* should be understood as the result of a crossover between local high-field transport effects and gate-controlled channel modulation rather than by the classical 1/*L_G_* scaling law alone. In particular, the reduced *g_m_*/*I_D_* of the 40 nm device suggests that the increased current drive in the ultrashort-channel regime is partially offset by degraded transconductance efficiency.

### 3.2. Small-Signal Frequency Characteristics

**Figure 10:**
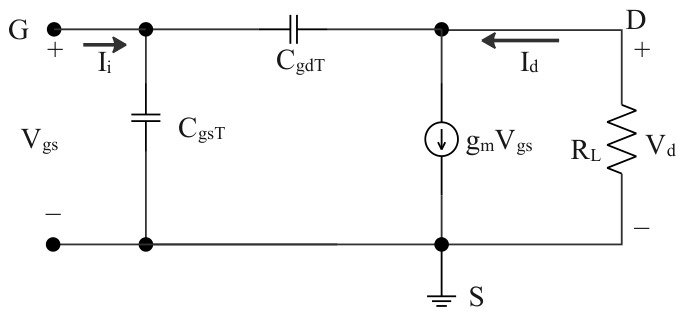
Simplified high-frequency small-signal equivalent circuit of the top-gate In_2_O_3_ FET.

To analyze the RF behavior of the simulated top-gate In_2_O_3_ TFTs under a controlled TCAD framework, a simplified high-frequency small-signal equivalent circuit was adopted, as shown in Figure 10. The devices with different gate lengths were biased in the saturation region at *V_DS_* = 6 V. The purpose of this equivalent-circuit analysis is to compare the intrinsic scaling trends of the capacitance network and transconductance rather than to provide a fully extrinsic circuit-level prediction.

Accordingly, several extrinsic non-ideal elements, including *R_S_*, *R_D_*, *R_DS_*, and *C_DS_*, were intentionally omitted in the present RF analysis so that the dependence of the intrinsic small-signal quantities on gate-length scaling could be examined more transparently. In this simplified circuit, *C_gsT_* denotes the total gate–source capacitance, *C_gdT_* denotes the total gate–drain capacitance, and *R_L_* is the load resistance, which was taken as 50 Ω for the high-frequency analysis. Based on the TCAD-extracted capacitance network, the relevant capacitance components were obtained for each gate length.

**Figure 11:**
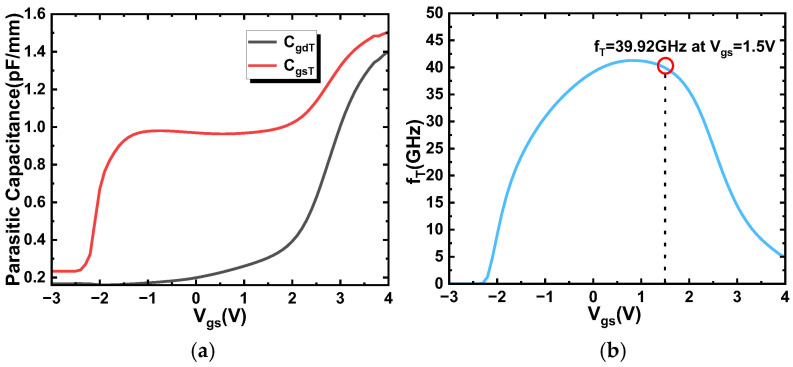
(**a**) *C_gdT_* and *C_gsT_* versus *V_GS_* at *V_D_* = 6 V; (**b**) *f_T_* versus *V_GS_* extracted from the small-signal capacitance network.

Taking the device with *L_G_* = 120 nm as an example, the extracted *C_gsT_* and *C_gdT_* are shown in Figure 11a. Combined with the transconductance data extracted from Figure 4d, the corresponding *f_T_* was then calculated according to the following formulas. Therefore, the RF discussion presented below should be understood as an intrinsic trend analysis within the present TCAD framework, while additional extrinsic parasitic and thermal effects remain beyond the scope of this work. A more comprehensive extrinsic RF treatment including additional resistive, thermal, and non-equilibrium effects will be valuable for future quantitative circuit-level studies.

According to KCL, we have:

(3) $$I_{i}\;=\;j\omega \left[C_{gsT}V_{gs}\;+\;j\omega C_{gdT}\left(V_{gs}\;-\;V_{d}\right)\right]$$

Similarly, the sum of currents at the output drain terminal is given by:

(4) $$\frac{V_{d}}{R_{L}}+g_{m}V_{gs}+j\omega C_{gdT}(V_{d}-V_{gs})=0$$

From the above two equations, we obtain:

(5) $$I_{i}=j\omega [C_{gsT}+C_{gdT}(\frac{1+g_{m}R_{L}}{1+j\omega R_{L}C_{gdT}})]V_{gs}$$

Since *ωR_L_C_gdT_* is typically much less than 1, the term *jωR_L_C_gdT_* can be neglected. Thus, the above equation simplifies to:

(6) $$I_{i}\;=\;j\omega \left[C_{gsT}\;+\;C_{gdT}\left(1\;+\;g_{m}R_{L}\right)\right]V_{gs}$$

The ideal load current is given by:

(7) $$I_{d}=g_{m}V_{gs}$$

The current gain is then given by:

(8) $$\left|\frac{I_{d}}{I_{t}}\right|=\frac{g_{m}}{2\pi f\left[C_{gsT}+C_{gdT}\left(1+g_{m}R_{L}\right)\right]}$$

When the current gain reaches unity, we obtain:

(9) $$f_{T}=\frac{g_{m}}{2\pi \left[C_{gsT}+C_{gdT}\left(1+g_{m}R_{L}\right)\right]}$$

**Figure 12:**
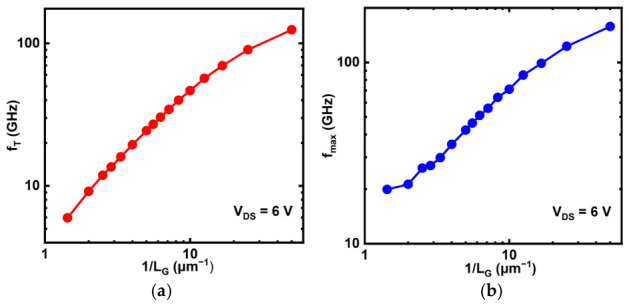
(**a**) Relationship of *f_T_* and (**b**) *f_max_* versus 1/*L_G_* of top-gate In_2_O_3_ FET.

The cutoff frequency (*f_T_*) is extracted from the simulation as the frequency increases to the 0 dB point of the current gain. The resulting *f_T_* of the laterally down-scaling devices at the *V_GS_* voltage of *g_m_max_* (see Figure 8b) is shown in Figure 12a. When the device with *L_G_* = 120 nm is biased at *V_DS_* = 6 V, its current gain cutoff frequency reaches 39.92 GHz at *V_GS_* = 1.5 V (where the *g_m_max_* occurs).

A gradual variation in the slope of the *f_T_* versus 1/*L_G_* relation is observed in the semi-log plot [see Figure 12a], which appears anomalous initially. This observation contrasts with the classical long-channel theoretical prediction for semiconductor FETs, where the cutoff frequency typically follows the relation:

(10) $$f_{T}=\frac{\mu_{0}(V_{GS}-V_{TH})}{2\pi {L^{2}}_{G}}$$

In the short-channel limit, the scaling relation transitions to *f_T_*∝1/*L_G_* [27]. Consequently, during the lateral scaling of FETs, a critical transition point—where the dependence shifts from *f_T_* ∝ 1/*L^2^_G_* to *f_T_* ∝ 1/*L_G_*—is expected, demarcating the long-channel and short-channel regimes. Notably, in contrast to the relatively ambiguous transition observed in the *f_T_* versus 1/*L_G_* plot, the *V_TH_* versus *L_G_* relation exhibits a distinct phase change at an *L_G_* of approximately 100 nm. The characteristic high carrier mobility of In_2_O_3_ fundamentally modifies the scaling behavior of its cutoff frequency. In the long-channel regime, the theoretical *f_T_* ∝ 1/*L^2^_G_* dependence stems from a constant mobility. However, for high-mobility materials like In_2_O_3_, carriers can more readily achieve and maintain velocity saturation under high lateral fields in shortened channels. This effect mitigates the degradation of carrier velocity with scaling, thereby blunting the expected transition to a pronounced *f_T_* ∝ 1/*L_G_* regime. Consequently, the long-channel scaling behavior persists observably into shorter gate lengths, resulting in a less distinct transition point in the *f_T_* versus 1/*L_G_* plot compared to materials with lower mobility.

The maximum oscillation frequency (*f_max_*) in this work is determined as the frequency at which the simulated unilateral power gain drops to 0 dB. Figure 12b presents the *f_max_* for the laterally scaled devices at the gate voltage corresponding to *g_m_max_*. For the device with *L_G_* = 120 nm and *V_DS_* = 6 V, its *f_max_* reaches 73.13 GHz at *V_GS_* = 1.5 V, where the *g_m_max_* occurs.

As shown in Figure 12b, the *f_max_* exhibits a non-monotonic variation with respect to 1/*L_G_*. In the regime of relatively long *L_G_*, *f_max_* exhibits a sluggish increase, which can be attributed within the present simplified framework to the relatively larger influence of the capacitance network and residual parasitic limitations. As *L_G_* enters the medium range, the device transitions into an approximate ideal scaling regime, where the enhancement of the intrinsic *f_T_* becomes the dominant factor, and *f_max_* thus approximates a linear increase:

(11) $$f_{max}\approx \frac{f_{T}}{2\sqrt{\left(R_{g}+R_{s}+\frac{R_{ch}}{g_{m}R_{s}})(g_{ds}+2\pi f_{T}C_{gd}\right)}}$$

When *L_G_* is further scaled down to the deep sub-micron regime, effects such as velocity saturation, the pronounced DIBL effect, and the relatively increasing influence of parasitic elements collectively act to slow down the growth rate of *f_max_* once again. This evolutionary process vividly illustrates the transition in dominance governing the high-frequency performance of heavily doped In_2_O_3_ FETs during lateral scaling: shifting first from parasitic limitations to intrinsic physical properties and finally to short-channel non-idealities.

**Figure 13:**
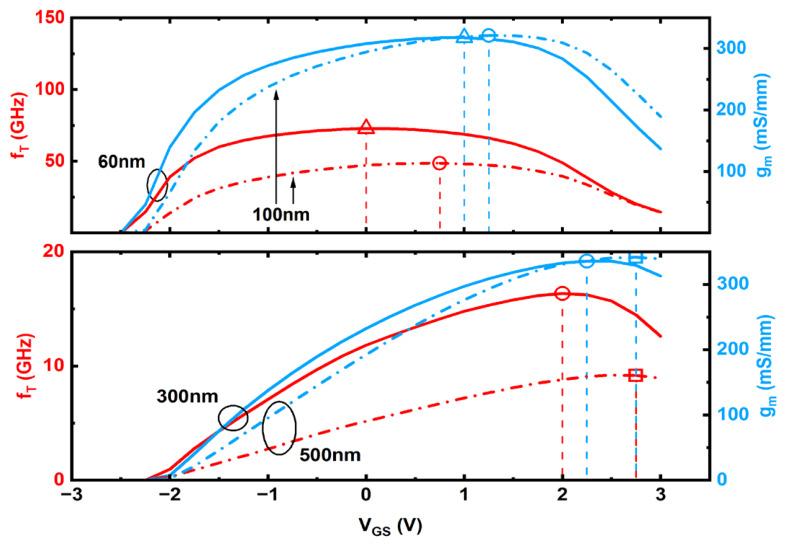
Relationship of *f_T_* and *g_m_* versus *V_GS_* at *V_DS_* = 6 V for the top-gate In_2_O_3_ FET with the given gate lengths. Hollow symbols with dropping lines indicate the maximum point of these relation curves.

Lastly, Figure 13 presents the relationship between the *f_T_* and the *V_GS_* for top-gate In_2_O_3_ FETs with scaled gate lengths at *V_DS_* = 6 V. The corresponding *g_m_* versus *V_GS_* is also included for reference. It can be observed that regardless of the *L_G_*, the variation trend of the *f_T_* with *V_GS_* closely resembles that of the *g_m_* with *V_GS_*:

(12) $$f_{T}=\frac{g_{m}}{2\pi C_{G}}$$

As evident from the Formula (12), given that the gate capacitance (*C_G_*) remains relatively constant, the variation in the *f_T_* is predominantly governed by the *g_m_*. Consequently, the trend of *g_m_* versus *V_GS_* is directly mirrored in the *f_T_* characteristic. For a top-gate In_2_O_3_ FET on the on-state, the gate capacitance is dominated by the gate oxide capacitance, while the channel capacitance exhibits minimal variation with *V_GS_*. Hence, after the device turns on, *C_G_* shows insignificant change with *V_GS_*, causing the variation in *f_T_* to primarily follow that of *g_m_*. Even with variations in *L_G_*, the Formula (12) remains valid provided that the device is on-state. Consequently, the curves depicting *f_T_* versus *V_GS_* and *g_m_* versus *V_GS_* are qualitatively similar across both long-channel and short-channel devices.

However, it is noteworthy that although the trends are similar, the *V_GS_* corresponding to the maximum *f_T_* (*f_T_max_*) and that corresponding to the *g_m_max_* may differ slightly. Several plausible reasons account for this discrepancy. Specifically, *f_T_* exhibits a stronger dependence on the effective gate overdrive voltage (*V_ov_* = *V_GS_* − *V_TH_*), whereas *g_m_* is more directly influenced by the drain current [28]. Parasitic resistances, such as source and drain resistances (*R_S_*, *R_D_*), also affect *f_T_* and *g_m_* to different extents [29,30]. Moreover, under pronounced short-channel effects, current saturation or velocity saturation can lead to a misalignment in the maximum positions of *g_m_* and *f_T_* [31].

## 4. Conclusions

This study investigated the lateral scaling characteristics of top-gate In_2_O_3_ TFTs with a 7 nm thick HfO_2_ gate dielectric and a centered gate configuration for *L_G_* ranging from 20 nm to 700 nm using a device simulator (Silvaco Atlas). This study focused on analyzing its short-channel effects, DC performance, and small-signal frequency response.

The results indicate that the critical *L_G_* for the transition from long-channel to short-channel behavior is approximately 100 nm. When *L_G_* ≤ 100 nm, short-channel effects cause a significant negative shift in the *V_TH_*, accompanied by a pronounced increase in the DIBL coefficient. When the *L_G_* is scaled below 40 nm, the *V_Dsat_* of the device even exceeds 8 V. For *L_G_* = 120 nm, the saturation drain current continues to increase with drain voltage rather than remaining constant, indicating the onset of evident short-channel non-idealities. This study further reveals that for a device with *L_G_* = 40 nm, the simulated *f_T_* reaches 90.24 GHz and the *f_max_* reaches 122.73 GHz. When *L_G_* is scaled to 20 nm, these values rise to 124.32 GHz for *f_T_* and 157.64 GHz for *f_max_*.

Overall, this work demonstrates through simulation the RF potential of top-gate In_2_O_3_ TFTs in the radio frequency domain, with *f*_T_/*f*_max_ values comparable to early-stage simulation or experimental results reported in the literature for other advanced RF technologies, such as GaN and Ga_2_O_3_ [32,33].

It should be noted that the present conclusions are drawn within a drift-diffusion-based TCAD framework with calibrated effective interface/transport parameters and a simplified intrinsic RF equivalent circuit. More comprehensive treatment of substrate dependence, tunneling, self-heating, and additional extrinsic parasitics will be valuable in future work.

## Figures and Tables

**Table 1:** Key fixed and calibration-guided parameters used in the TCAD simulations.

Parameter Name	Value
Work function of Ni	5.1 eV
Electron doping concentration in the indium oxide material	2.5 × 10^20^ cm^−3^
Fixed charges	1 × 10^10^ cm^−2^
In_2_O_3_ material grouping	Semiconductor
In_2_O_3_ electron affinity	3.7 eV
In_2_O_3_ bandgap at 300 K	3.6 eV
In_2_O_3_ relative permittivity	8.5
In_2_O_3_ electron mobility	120 cm^2^/(V·s)
In_2_O_3_ effective density of states in conduction band at 300 K	6 × 10^18^ cm^−3^
In_2_O_3_ effective density of states in valence band at 300 K	6 × 10^18^ cm^−3^
In_2_O_3_ electron saturation velocity	1 × 10^7^ cm/s
Other materials’ parameters	Silvaco default
SRH lifetime	10 ns

## Data Availability

The original contributions presented in this study are included in the article. The data that support the findings of this study are available from the corresponding author upon reasonable request.

## Supplementary Materials

The following supporting information can be downloaded at: https://www.mdpi.com/article/10.3390/mi17050567/s1, Figure S1: Additional consistency check using the top-gate In_2_O_3_ TFT structure reported in Ref. [10] with the calibration-guided parameters and physical models used in this work; Figure S2: Comparison of output and transfer characteristics for devices with sapphire, SiO_2_, and SiO_2_-on-Si substrates under otherwise identical device geometry and physical-model settings. (a) Sapphire versus SiO_2_ substrate [10]. (b) Sapphire versus SiO_2_-on-Si substrate [13]. The gate length is 120 nm, and the bias conditions are kept the same as those used for the representative device in the main manuscript.
