# Supplementary material for: Numerical Investigation of Short-Channel Effects and RF Performance in Top-Gate In2O3 Thin-Film Transistors
Source: Micromachines (Basel). 2026 May 2;17(5):567. doi: 10.3390/mi17050567 (PMC13209046; doi:10.3390/mi17050567)
Supplement: Supplementary file 1 [file micromachines-17-00567-s001.zip › micromachines-4259285-supplementary.pdf]

Supplementary Material

Supplementary Material for "Numerical Investigation of Short-Channel Effects and RF Performance in Top-Gate In_2O_3 Thin-Film Transistors"

Hanbo Xu [1,†], Mingyang Zhu [1,†], Zeen Fang [1] and Lei Zhang [1,*]

[1] College of Mechanical and Electrical Engineering, Central South University, Changsha 410083, China.
† Hanbo Xu and Mingyang Zhu are co-first authors.
* Correspondence: zhangl207@csu.edu.cn

1. Additional Consistency Check Using the Top-Gate Device in Ref. [10]

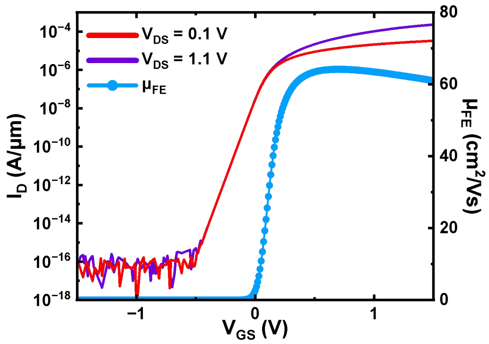

Figure S1. Additional consistency check using the top-gate In_2O_3 TFT structure reported in Ref. [10] with the calibration-guided parameters and physical models used in this work.

To further evaluate the consistency of the calibration-guided parameter set used in this simulation work, we transferred the calibration parameters and physical models described in Sections 2.1 and 2.3 of the main manuscript to the device structure of Ref. [10]. It should be clarified that, when performing this consistency check using Ref. [10], we rebuilt a 3D model according to the device structure reported in Ref. [10]; therefore, the device structure used here is not Fig. 1(a) of this work, but Fig. 1(a) of Ref. [10] ("In-Situ Surface Energy Engineering for ALD-Derived Highly Reliable Top-Gate In_2O_3 Thin-Film Transistors").

After the device structure was defined and all parameters and physical models were set, the I_D–V_{GS} curves at V_{DS} = 0.1 V and V_{DS} = 1.1 V, as well as the extracted field-effect mobility versus V_{GS}, were extracted from the simulated device following the condition specified in Fig. 2(a) of Ref. [10] (i.e., treatment time = 0 s), as shown in Fig. S1 of the Supplementary Material. A comparison of the simulated and experimental results reveals that the trend and order of magnitude of I_D are similar. The simulated field-effect mobility exhibits a trend comparable to the experiment; however, its decay with gate voltage is slower and lies between the results shown in Figs. 1(a) and 1(b) of Ref. [10].

The remaining discrepancy is likely related to process-specific details and interface conditions that are not fully captured in the present device-level TCAD framework. Nevertheless, the comparable trend and order of magnitude support the reasonableness of the calibration-guided parameter set.

2. Results on SiO_2 Substrate and SiO_2-on-Si Substrate

As mentioned in the main manuscript, the purpose of this work is to investigate a new top-gate In_2O_3 TFT structure on a sapphire substrate, rather than to replicate the structures reported in Refs. [10] and [13]. To provide a more complete investigation, additional

simulations were carried out in which the sapphire substrate of the present device was replaced by the SiO_2 substrate and the SiO_2-on-Si substrate mentioned in Refs. [10] and [13], while all other conditions remained unchanged. A comparison of the simulation results is shown in Fig. S2.

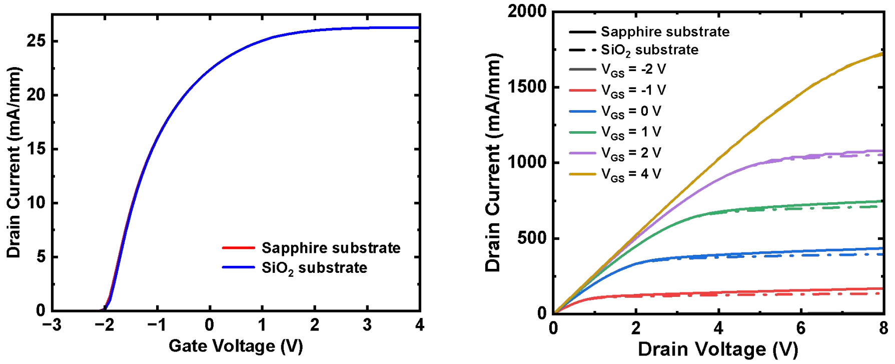

(a)

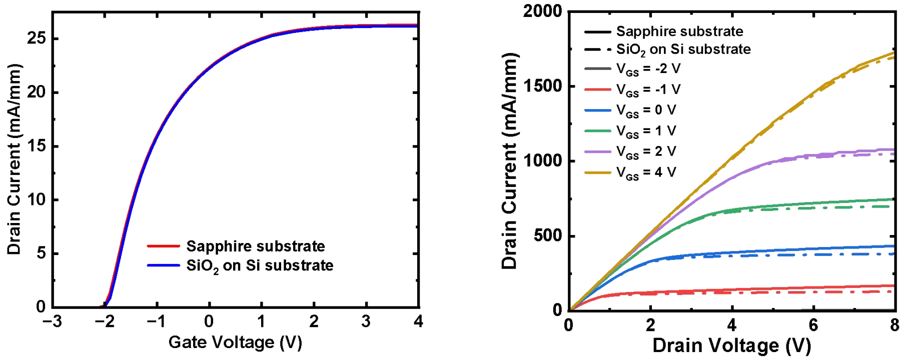

(b)

Figure S2. Comparison of output and transfer characteristics for devices with sapphire, SiO_2, and SiO_2-on-Si substrates under otherwise identical device geometry and physical-model settings. (a) Sapphire versus SiO_2 substrate [10]. (b) Sapphire versus SiO_2-on-Si substrate [13]. The gate length is 120 nm, and the bias conditions are kept the same as those used for the representative device in the main manuscript.

Under the present top-gate configuration and electrically passive substrate-boundary conditions, replacing sapphire with SiO_2 or SiO_2-on-Si produces only minor changes in the simulated output and transfer characteristics. This indicates that the main scaling trends discussed in the manuscript are dominated by the top-gate/channel electrostatics rather than by the supporting substrate.
